# Characterization of taxonomically restricted genes in a phylum-restricted cell type

**DOI:** 10.1186/gb-2009-10-1-r8

**Published:** 2009-01-22

**Authors:** Sabine Milde, Georg Hemmrich, Friederike Anton-Erxleben, Konstantin Khalturin, Jörg Wittlieb, Thomas CG Bosch

**Affiliations:** 1 Zoological Institute, Christian-Albrechts-University Kiel, Olshausenstr. 40, 24098 Kiel, Germany

## Abstract

**Background:**

Despite decades of research, the molecular mechanisms responsible for the evolution of morphological diversity remain poorly understood. While current models assume that species-specific morphologies are governed by differential use of conserved genetic regulatory circuits, it is debated whether non-conserved taxonomically restricted genes are also involved in making taxonomically relevant structures. The genomic resources available in *Hydra*, a member of the early branching animal phylum Cnidaria, provide a unique opportunity to study the molecular evolution of morphological novelties such as the nematocyte, a cell type characteristic of, and unique to, Cnidaria.

**Results:**

We have identified nematocyte-specific genes by suppression subtractive hybridization and find that a considerable portion has no homologues to any sequences in animals outside *Hydra*. By analyzing the transcripts of these taxonomically restricted genes and mining of the *Hydra magnipapillata *genome, we find unexpected complexity in gene structure and transcript processing. Transgenic *Hydra *expressing the green fluorescent protein reporter under control of one of the taxonomically restricted gene promoters recapitulate faithfully the described expression pattern, indicating that promoters of taxonomically restricted genes contain all elements essential for spatial and temporal control mechanisms. Surprisingly, phylogenetic footprinting of this promoter did not reveal any conserved *cis*-regulatory elements.

**Conclusions:**

Our findings suggest that taxonomically restricted genes are involved in the evolution of morphological novelties such as the cnidarian nematocyte. The transcriptional regulatory network controlling taxonomically restricted gene expression may contain not yet characterized transcription factors or *cis*-regulatory elements.

## Abstract 2

Computational and functional genomic analyses in Hydra magnipapillata suggest that taxonomically-restricted genes are involved in the evolution of morphological novelties such as the cnidarian nematocyte

## Background

Cnidaria represent the simplest animals at the tissue grade of organization. In order to catch prey, cnidarians have evolved a unique "high-tech cellular weaponry" [1] - the stinging cells (cnidocytes, nematocytes) - single cells able to shoot structures at their target and inject toxic substances into it. Nematocytes are unique to and present in all species of the phylum Cnidaria. Different phylogenetic lines have different nematocyte types [2,3]. Evolution of cnidarian families appears to be accompanied by expansion of the nematocyte repertoire [4]. In *Hydra*, four types of nematocytes can be distinguished based on the distinct morphology of the nematocyte capsule: stenotele, desmoneme, holotrichous isorhiza and atrichous isorhiza. Previous work [5,6] has identified unusually short proteins with a collagen-related domain (minicollagens) as major constituents of the nematocyst capsule wall. Intermolecular disulfide bonds between the cysteine-rich domains of these minicollagens and an additional capsule protein, NOWA, are thought to stabilize the capsule wall [7]. The spines inside the capsules contain spinalin, another protein unrelated to any protein in other animals [8].

How novel morphological structures evolve is an open and important question. One currently popular view is that since many genes are shared throughout the animal kingdom, animal diversity is largely based on differential use of conserved genes and regulatory circuits [9-11]. However, all genome and expressed sequence tag (EST) projects to date in every taxonomic group studied so far have uncovered a substantial amount of genes that are without known homologues [12,13]. A previous study [13] has discovered that a family of such taxonomically restricted 'orphan' genes plays a significant role in controlling phenotypic features referred to as species-specific traits in the genus *Hydra*. Thus, morphological diversity in closely related species may be generated through changes in the spatial and temporal deployment of genes that are not highly conserved across long evolutionary distances [13].

We here have chosen an unbiased comparative approach based on suppression subtractive hybridization (SSH) to identify additional nematocyte-specific genes in *Hydra*. Among those detected, a considerable portion has no homologues in animals outside *Hydra*. Since they are exclusively restricted to the phylum Cnidaria, they are considered as 'orphans' or 'taxonomically restricted genes' (TRGs) [13-16].

Analysis of these TRGs indicates striking complexity in their genomic organization and transcript processing. In order to understand how such TRGs are regulated, we generated transgenic polyps that express green fluorescent protein (GFP) under control of one of the TRG promoters. Transgenic *Hydra *recapitulate faithfully the previously described expression pattern, indicating that the promoter contains all elements essential for spatial and temporal control mechanisms. Surprisingly, phylogenetic footprinting of this promoter did not reveal any conserved *cis*-regulatory elements. This may indicate that the transcriptional regulatory network controlling TRG expression may contain not yet characterized transcription factors or *cis*-regulatory elements.

Our data provide a detailed genomic description of several taxonomically restricted genes in a basal metazoan, and functional evidence that TRGs are integrated in transcriptional regulatory networks to form functional signaling cascades.

## Results

### Identification of taxonomically restricted genes expressed in nematocytes

In order to isolate not yet identified genes potentially involved in nematocyte differentiation, we made use of the sf-1 mutant strain of *H. magnipapillata*, which has temperature-sensitive interstitial stem cells [17]. Interstitial cells are located between the ectodermal epithelial cells and contain both germline and somatic components, giving rise to all nerve cells, gland cells and nematocytes [18]. Treatment for a few hours at the restrictive temperature (28°C) induces quantitative loss of the entire interstitial cell lineage, including nematocytes from the ectodermal epithelium [19].

**Figure 1:**
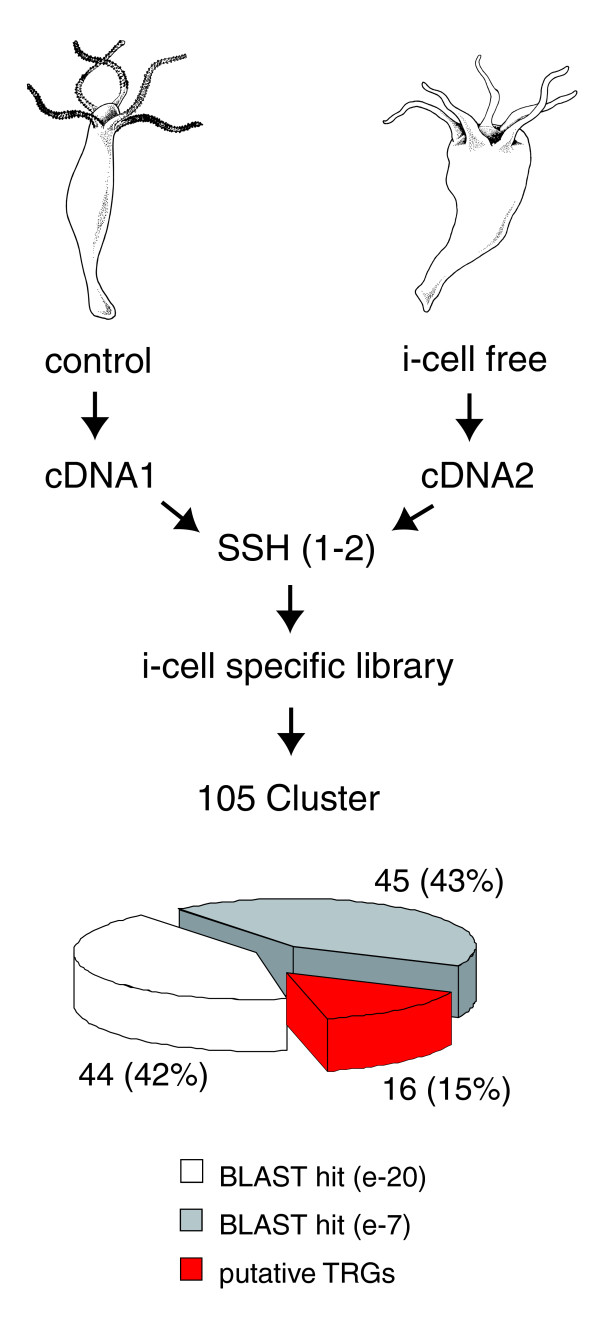
**Identification of interstitial cell lineage-specific genes in *Hydra *by suppression subtractive hybridization (SSH)**. *H. magnipapillata *(strain sf-1) cDNA was used as tester and cDNA of interstitial cell free *H. magnipapillata *(sf1) as driver to generate a library enriched for transcripts of the interstitial cell lineage. BLASTx analysis could group 105 EST-contig sequences into three categories of homology: 45 sequences (43%) had strong similarities (e-value < 1e-20) to known metazoan proteins; 44 sequences (42%) had low e-values (>1e -7); 16 sequences (15%) had no homologues in the NCBI protein database, representing genes putatively restricted to the genus *Hydra*.

To identify genes that are transcriptionally active in differentiating nematocytes, we compared transcriptomes of control and nematocyte-free *H. magnipapillata *by SSH of cDNAs. As shown schematically in Figure 1, subtractive hybridization resulted in a cDNA library enriched for interstitial stem cell lineage-specific transcripts. Sequencing of 2,500 clones revealed 105 consensus contig sequences that could be grouped by BLASTx analysis into three different categories of homology (Figure 1). One set (45 sequences; 43%) had strong similarities (e-value < 1e-20) to known metazoan proteins. The second set (44 sequences; 42%) had low e-values (>1e -7) and represents genes related but not identical to previously identified metazoan genes. The third set (16 sequences; 15%) had no homologues in the National Centre for Biotechnology Information (NCBI) protein database (Figure 1), representing, therefore, genes putatively restricted to *Hydra *or Hydrozoa. Further sequence analysis of these 16 contigs revealed that some of them (contigs 049 and 129 as well as 035 and 109) represent fragments of the same primary transcript. Thus, the approach resulted in identification of a total of 14 genes without significant homology.

**Figure 2:**
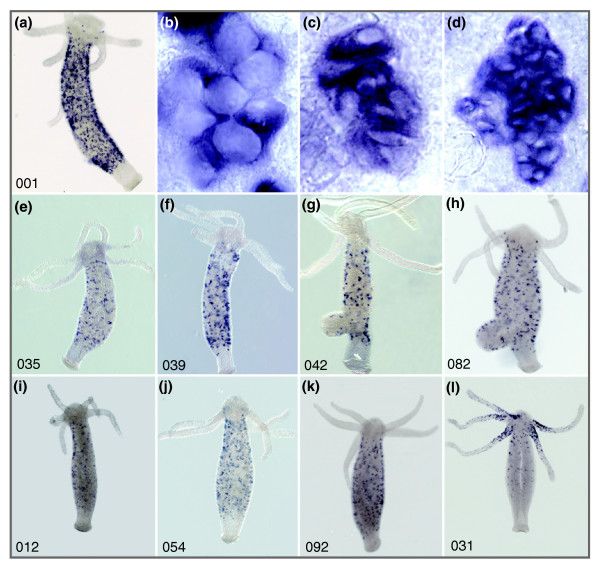
**Expression of taxonomically restricted genes identified in the suppression subtractive hybridization screening in *Hydra *nematocytes**. Whole mount *in situ *hybridization of nine TRG sequences represent transcripts expressed exclusively in differentiating nematocytes. **(a-h) **Five transcripts show expression in all types of differentiating nematocytes: *nb001 *(a), *nb035 *(e), *nb039 *(f), *nb042 *(g), *nb082 *(h). **(b-d)** Magnifications of nematoblast nests: stenotheles (b); izorhiza (c); desmonemes (d). **(i-k) **Three TRGs are expressed only in isorhiza and desmonemes: *nb012 *(i); *nb054 *(j); *nb092 *(k). **(l) **One TRG, *nb031*, is exclusively expressed in stenoteles predominantly at the base of tentacles.

Next, we analyzed the expression of these putative TRGs by whole mount *in situ *hybridization. Out of the 14 genes, 9 represent transcripts expressed exclusively in differentiating nematocytes. While five of them (Figure 2a-h; *nb001*, *nb035*, *nb039*, *nb042*, *nb082*) show expression in all types of differentiating nematocytes, three genes (Figure 2i-k; *nb012*, *nb054*, *nb092*) are expressed only in isorhiza and desmonemes. One gene (*nb031*; Figure 2l) is exclusively expressed in stenoteles, predominantly at the base of tentacles.

**Figure 3:**
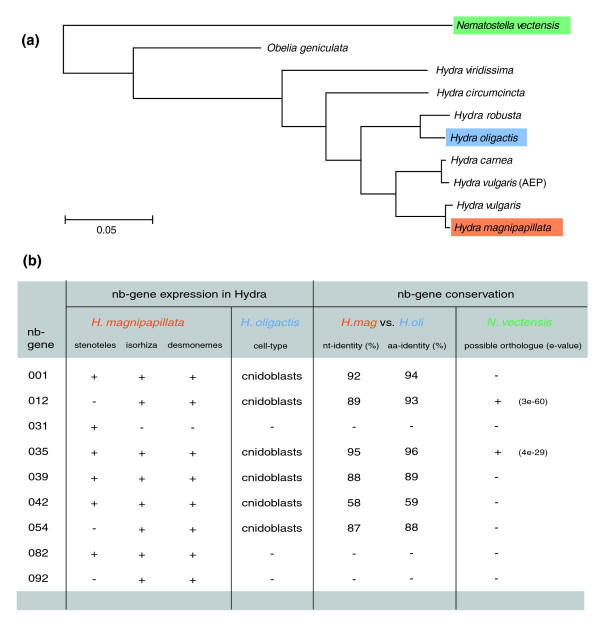
**Comparative expression analysis of taxonomically restricted genes in different types of developing nematocytes**. **(a) **Phylogenetic relationships in the genus *Hydra*; colors indicate the examined species referred to in the table in (b); phylogenetic tree modified from [20]. **(b) **TRG expression in developing nematocytes in two different *Hydra *species (*H. magnipapillata *and *H. oligactis*) as well as conservation of corresponding TRGs between the two species and possible othologuous sequences in the distantly related anthozoan sea anemone *Nematostella vectensis*. aa, amino acid; nt, nucleotide.

To investigate whether the identified genes were restricted to the species *H. magnipapillata *or are also present in other *Hydra *species (Figure 3a), we analyzed their expression in the related *Hydra oligactis *[20]. Figure 3b indicates that genes *nb012*, *nb035*, *nb039*, *nb042 *and *nb054 *give a strong *in situ *hybridization signal in differentiating nematocytes in both *H. magnipapillata *and *H. oligactis*, representing, therefore, genes putatively restricted to the genus *Hydra*. TRGs found to be expressed in nematocytes in both species share high sequence similarity at the nucleotide and amino acid levels. Figure 3b also indicates that transcripts for *nb031*, *nb082*, and *nb092 *cannot be detected in *H. oligactis*, representing, therefore, genes putatively restricted to the species *H. magnipapillata*. Interestingly, screening the genome of the anthozoan sea anemone *Nematostella vectensis *provided evidence for the presence of at least two of the above-described nematocyte-specific TRGs in this distantly related cnidarian (Figure 3b). Thus, these genes seem to be present in many classes of the phylum Cnidaria but absent in other metazoan taxa. Therefore, such genes might be considered 'cnidaria-specific'.

### Characterization of taxonomically restricted genes expressed in nematocytes

#### A novel family of minicollagen proteins originates from one genomic locus

**Figure 4:**
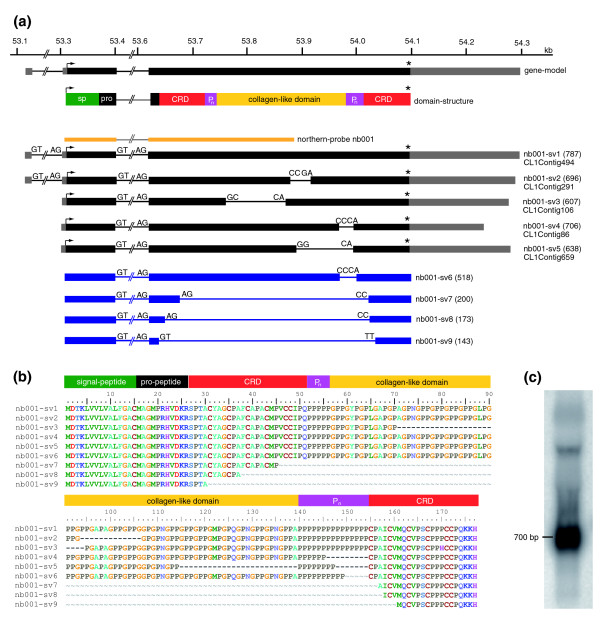
**Genomic organization and alternative transcripts of *nb001*/*minicollagen***. **(a) **Mapping of *nb001 *EST-contigs (*nb001*-*sv1 *to *nb001*-*sv5*; black) and amplified PCR products (*nb001*-*sv6 *to *nb001*-*sv9*; blue) to the corresponding genomic locus (*H. magnipapillata *genomic scaffold NW_002161526). *nb001 *transcripts encode a protein with a signal peptide (sp; green), pro-peptide (pro; black) and a collagen-like domain (yellow) flanked by two praline repeats (P_n_; magenta) and two cystein-rich-domains (CRD; red). **(b) **Alignment of the amino acid sequences of predicted splice variants. **(c) **Northern blot indicates the presence of *nb001 *transcripts corresponding to most of the predicted splice variants. Probe for hybridization corresponds to exon 2 and the first half of exon 3 (yellow line in (a)). Asterisks indicate stop codons.

Detailed analysis of the gene *nb001 *revealed that it encodes a novel member of the minicollagen family of proteins containing the previously reported [5,21,22] structural features such as a signal peptide, propetide, cystein rich domain, and a proline-repeat flanked collagen-like domain (Figure 4a). In a recent review [4] the protein encoded by *nb001 *was referred to as 'minicollagen 6'. At the nucleotide level, *nb001 *shares no similarity to previously published [5,22] minicollagens.

Analysis of *nb001 *transcripts in the EST data bank and the corresponding genomic locus uncovered five different splice variants (Figure 4a, *nb001*-*sv1 *to *nb001*-*sv5*: CL1Contig4, CL1Contig3, CL1Contig2, CL1Contig1 and CL1Contig5, respectively). In addition, by PCR amplification we could identify four more splice variants (*nb001*-*sv6 *to *nb001*-*sv9*; Figure 4a). Interestingly, while the first two introns are spliced by conventional splicing sites (GT/AG), additional variants of the transcripts are generated by processing of exon 3. As a result of this process, which may use unconventional 'splicing' sites, various regions of exon 3 are removed.

The resulting nb001 predicted proteins (Figure 4b) indicate domain length variations of the collagen-like domain as well as the proline and cysteine repeats. In contrast to previously reported minicollagens [5,22], all nb001 variants described here have 19-27 Gly-X-Y repeats instead of 12-16, resulting in an expanded collagen-like domain (Figure 4b). Other nb001 variants are characterized by a shortened praline repeat following the collagen-like domain. Three variants (*nb001*-*sv7 *to *nb001*-*sv9*) lack both the collagen-like domain and the proline repeats. These variants contain only a single cystein rich domain with an altered cysteine pattern - (CXXX)_7_-CC, (CXXX)_5_-CC or (CXXX)_2_-CC - instead of the conserved (CXXX)_4_-CC. Northern blot analysis (Figure 4c) shows a strong signal at around 700 bp, indicating the presence of *nb001 *transcripts corresponding to most of the predicted variants.

#### *Spinalin*, a previously identified nematocyte-specific gene is a splice variant derived from a complex genetic locus

**Figure 5:**
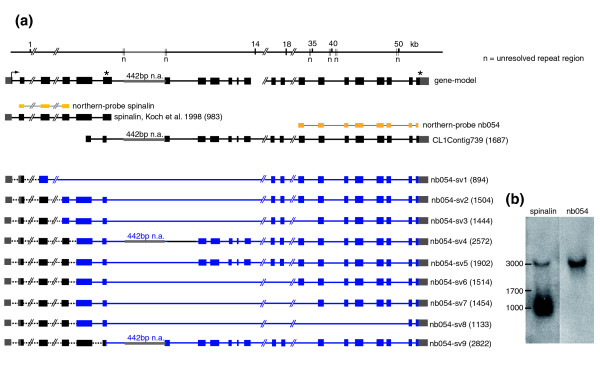
**Genomic organization and alternative transcripts of *nb054*/*spinalin***. **(a) **Mapping of *spinalin *and *nb054 *alternative transcripts to the corresponding genomic locus (*H. magnipapillata *genomic scaffold NW_002161446). The resulting gene-model shows two alternatively used stop codons indicated by asterisks. Since this genomic region is rich in AT repeats (n), some sequence areas remain unresolved and, therefore, the final number of *nb054*-specific exons remains to be determined. **(b) **Northern blot analysis with *spinalin *and *nb054 *specific probes (yellow lines in (a)).

Genomic analysis of TRG *nb054 *(Figure 5a) revealed that the corresponding 50 kb spanning genomic locus contains the gene *spinalin*, which was previously reported [8] to be involved in spine development of nematocysts. Sequence analysis confirmed by PCR amplification studies revealed that *spinalin *and *nb054 *are, in fact, encoded by a single gene and, therefore, must be considered as splice variants. While the first six exons encode the previously identified *spinalin*, splicing within the 6th exon leads to much longer transcript variants containing the first 6 exons plus an additional 2-16 exons, resulting in a large number of differentially spliced transcripts of about 3,000 bp (Figure 5a). The short 983 bp transcript encoding spinalin is produced by alternative splicing and usage of the resulting stop codon within exon 6. Since this genomic region is rich in AT repeats (Figure 5a), some sequence areas encoding the TRG *nb054 *remain unresolved and, therefore, the final number of *nb054*-specific exons remains to be determined. Northern blot analysis with *spinalin*- and *nb054*-specific probes (Figure 5b) revealed three distinct signals of about 1, 1.7 and 3 kb corresponding to the predicted *spinalin *and *nb054 *transcripts.

#### Gene duplication contributes to the complexity of nematocyte-specific gene families

**Figure 6:**
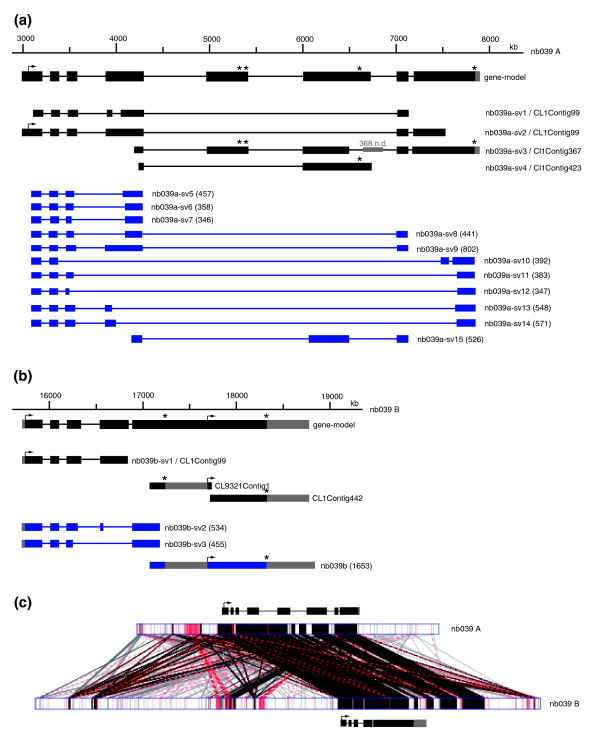
**Genomic organization and alternative transcripts of *nb039 *loci A and B**. **(a) **Genomic organization and alternative transcripts of *nb039 *locus A. **(b) **Genomic organization and alternative transcripts of *nb039 *locus B. **(c) **Comparative sequence analysis of both loci. Note that the gene-encoding part of nb039-A and nb039-B is highly conserved but flanked by stretches of non-conserved genomic sequences. Asterisks indicate stop codons.

The TRG *nb039 *has blast hits to two distinct but similar genomic contigs (NW_002158707, NW_002162805), which we named nb039-A and nb039-B (Figure 6a,b). Corresponding ESTs could be grouped into two independent sets of EST contigs, which are identical to the respective genomic locus and represent several different splice variants. Additionally, we were able to amplify 11 more partial splice variants for nb039-A and three more partial splice variants for nb039-B. From the locus nb039-A, two splice variants use alternative 3' untranslated regions (UTRs; *nb039a*-*sv4*/CL1Contig423, *nb039a*-*sv10*) due to early stop codons, which most likely were inserted by alternative splicing. Comparison of the exon/intron distribution pattern in the 5' adjacent region of nb039-A and nb039-B (Figure 6a,b) indicates striking structural similarity. A comparative sequence analysis of both loci (Figure 6c) provided evidence that they are the result of a gene duplication event since the gene-encoding part of nb039-A and nb039-B is highly conserved but flanked by stretches of non-conserved genomic sequences.

**Figure 7:**
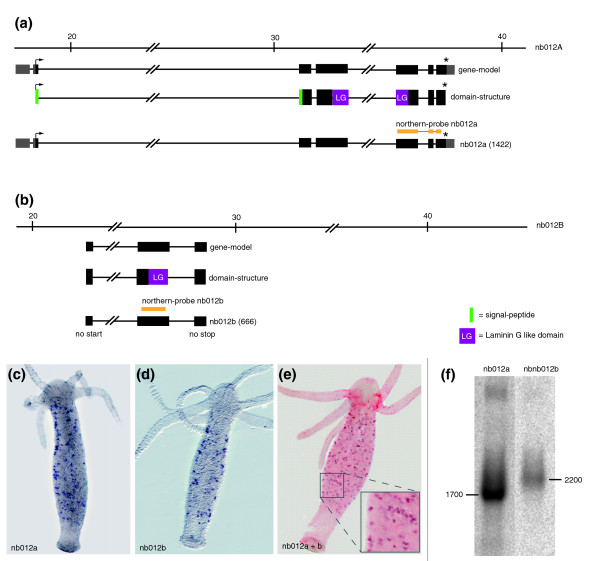
**Genomic organization of *nb012 *loci A and B**. **(a) **Genomic organization of *nb012 *locus A. **(b) **Genomic organization of *nb012 *locus B. **(c, d) **Expression of *nb012a *and *nb012b *in nematoblasts. **(e) **Double *in situ *hybridization using digoxigenin- and biotin-labeled probes for *n012a *and *nb012b*, respectively. As indicated in the higher magnification inset, both transcripts are co-localized in the same set of nematoblasts. **(f) **Northern blot analysis using *nb012a*- and *nb012b*-specific probes (yellow lines in (a, b)). Asterisks indicate stop codons.

A second example of a putative gene duplication event in a TRG gene expressed in nematocytes was discovered when analyzing the genomic locus of *nb012*. As shown in Figure 7a, this gene consists of seven exons corresponding to one EST contig (*nb012a*/CL243Contig1). The full-length transcript of this EST-contig contains a laminin-G-like domain located on exons 4 and 5. Screening the available *Hydra *EST collections revealed a second partial transcript with a laminin G-like domain with a sequence related but not identical to *nb012a*. We termed this transcript *nb012b *(Figure 7b). The available genome assembly suggests that this second partial transcript is encoded within the gene encoding *nb012a*. PCR based analysis, however, did not provide evidence for a transcript containing sequences of both *nb012a *and *nb012b*. Since a more informative re-assembly of the *nb012 *locus is currently not possible because of limited sequence data, we assume but cannot prove that *nb012a *and *nb012b *represent gene duplication events. *In situ *hybridization using *nb012a*- and *nb012b*-specific probes indicated (Figure 7c-e) that *nb012b *indeed represents a gene co-expressed with *nb012a*. The low level of sequence similarity in the probes used for the *in situ *hybridization analysis excluded the possibility of cross-hybridization. Double *in situ *hybridization confirmed that both genes are spatially and temporarily co-expressed in the same set of nematocytes (Figure 7e). Furthermore, Northern blot analysis (Figure 7f) using the *nb012a*- and *nb012b*-specific probes indicated the presence of two independent transcripts of about 1,700 and 2,200 bp, respectively. This supports the view that both genes are located on different genomic loci.

#### Sharing 3' UTRs in some nematocyte specific genes indicates common regulation of different splice variants

**Figure 8:**
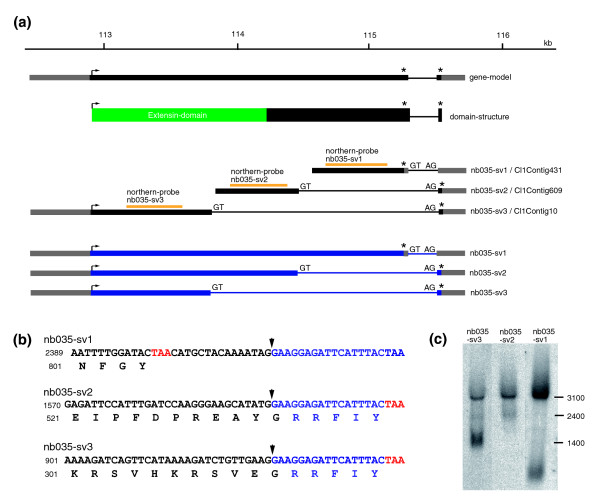
**Genomic organization and alternative transcripts of *nb035***. **(a) **Genomic organization and splice variants of *nb035 *(*H. magnipapillata *genomic scaffold NW_002151021). **(b) **As a result of alternative splicing three proteins with different carboxy-terminal sequences are encoded. **(c) **Northern blot analysis using probes specific for the three splice variants (yellow lines in (a)). Asterisks indicate stop codons.

Analyzing the genomic locus encoding TRG *nb035 *revealed a gene consisting of two exons (Figure 8a). While the first exon encodes a large open reading frame of 2,347 bp, the second exon is short and represents mainly 3' UTR. Three partial contigs (CL1Contig431, CL1Contig609, CL1Contig10) could be identified in the EST project and map to this locus. Rapid amplification of cDNA ends (3' and 5' RACE; Figure 8a) revealed that *nb035 *encodes three distinct splice variants (*nb035*-*sv1 *to *nb035*-*sv3*) that share a common 3' UTR. While the stop codon of *nb035*-*sv1 *is located at the end of the first exon, the stop codons for *nb035*-*sv2 *and *nb035*-*sv3 *are located in exon 2 (Figure 8a,b). As a result, corresponding proteins differ in their carboxy-terminal parts. Exon 1 encodes an extensin-related domain, which is altered in *nb035*-*sv3*. Northern blot analysis using probes specific for the three splice variants (Figure 8c) shows three distinct signals of 1,400, 2,400 and 3,100 bp, respectively.

### How are taxonomically restricted genes regulated?

#### The 1 kb upstream region of *nb001 *lacks any conserved transcription factor binding sites

How are genes that lack sequence similarity to known genes regulated? In an attempt to unravel the transcriptional regulatory network controlling expression of a TRG, we analyzed the *nb001 *5' flanking sequence. To identify the 5' regulatory sequence, we used the *H. magnipapillata *genome data deposited at NCBI. Since *nb001 *is expressed in a seemingly identical manner across species borders (Figure 3b), we reasoned that sequences important for control of *nb001 *expression were strongly conserved at the nucleotide level, since their potential for mutation is constrained by their function. As described previously [23], such evolutionarily conserved *cis*-regulatory elements can be identified by phylogenetic footprinting.

**Figure 9:**
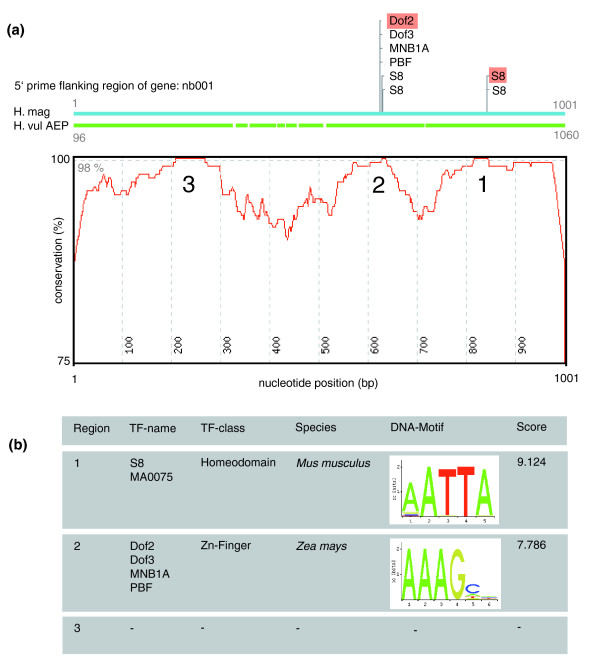
**Analysis of the *nb001 *promoter by phylogenetic footprinting**. **(a) **Conservation profile of *H. vulgaris *strain AEP and *H. magnipapillata nb001 *5' flanking regions. Three regions (1-3) exceed the conservation cut-off (98%) used for transcription factor (TF) binding site prediction. **(b) **Top-scoring motifs resulting from computational transcription factor binding site prediction (Consite).

Approximately 1 kb of 5' flanking sequence of the *nb001 *gene was analyzed from *H. magnipapillata *(strain 105) and closely related *H. vulgaris *(strain AEP) using the previously described ConSite platform [23]. As shown in Figure 9a, the 5' flanking regions are of unexpected high overall identity, with three regions, named regions I, II and III (Figure 9a), nearly identical between the two different species. These regions were subjected to conserved transcription factor binding site prediction (Figure 9b). As *Hydra *has an AT-rich genome composition, several cycles of analysis were performed with increasing transcription factor score thresholds, thus modulating the stringency of the sequence analysis. However, apart from AT-rich stretches, no conserved and informative binding motif remained detectable (Figure 9b).

#### The 1 kb upstream region of *nb001 *is essential and sufficient for correct expression *in vivo*

**Figure 10:**
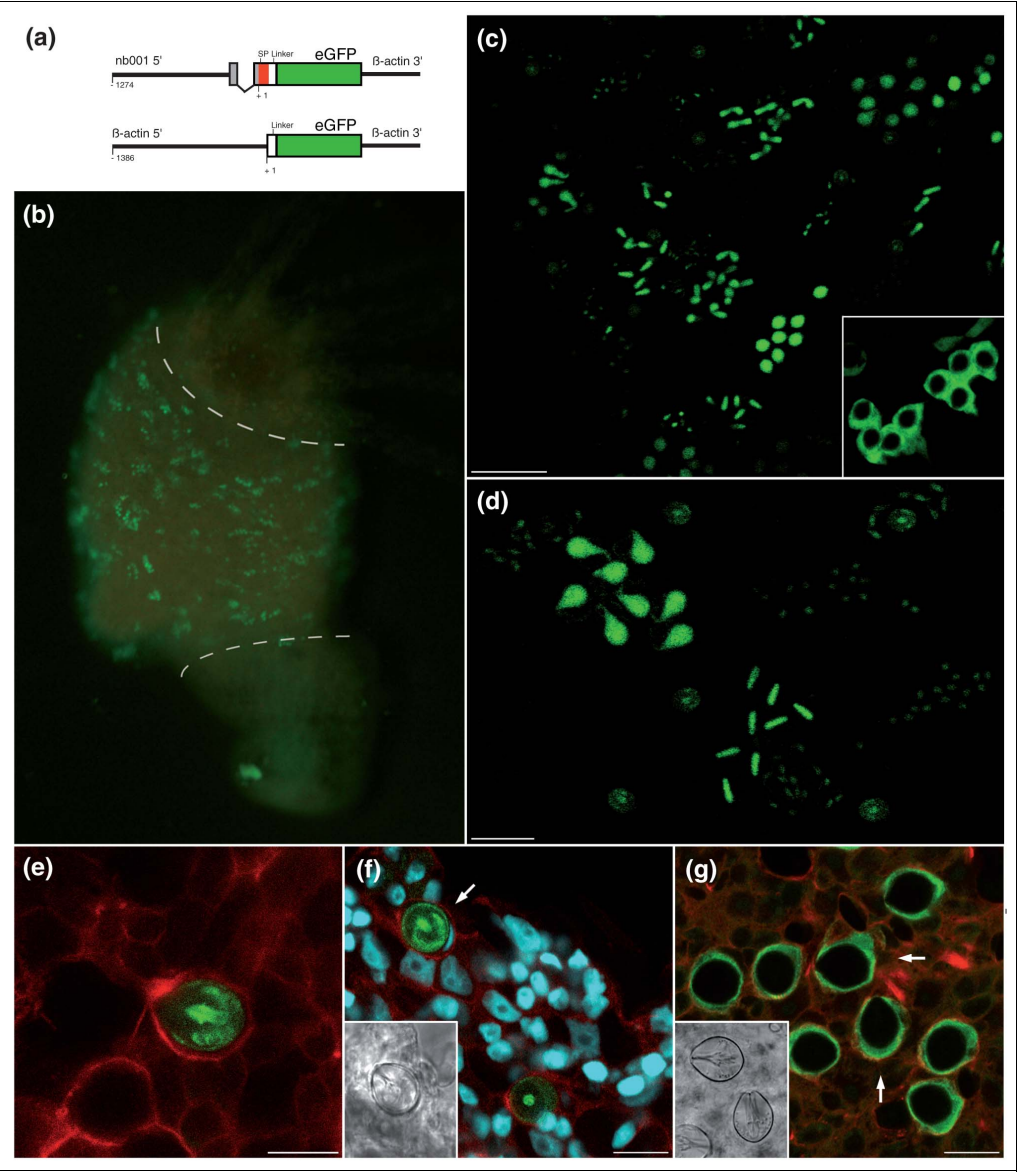
**Functional analysis of the *nb001 *promoter using transgenic polyps**. **(a) **Expression constructs for generation of transgenic *Hydra *(SP, signal peptide). **(b) **Polyp with eGFP-expressing differentiating nematocytes. Note that the *nb001 *driven eGFP expression recapitulates the nb001 expression shown in Figure 2a. As indicated by the dashed lines, expression is located only in the bodycolumn and not in the head or foot. **(c) **Confocal analysis of polyp containing eGFP-expressing nematocytes reveals that the promoter drives expression of eGFP in all four types of nematocytes; scale bar, 50 μm. The inset indicates control transgenic nematocytes with eGFP expression under control of actin promoter. **(d) **Transgenic nematocytes provide *in vivo *evidence for the view [28] that differentiating nematocytes undergo several rounds of synchronous cell division and remain connected to each other by cytoplasmic bridges to form nests of 4, 8 or more cells; scale bar, 25 μm. **(e) **Tentacle of transgenic polyp in which many but not all nematocytes are expressing eGFP. Confocal analysis. Green, eGFP protein; red, actin filaments; scale bar, 10 μm. **(f) **Confocal analysis of transgenic stenotele (arrow) in the gastric region showing eGFP protein localized within the capsule wall and tubule of the nematocyst. Red, actin filaments; scale bar, 15 μm. **(g) **Control transgenic polyp with eGFP expression under control of actin promoter. Note that eGFP is localized within the cytoplasm and the nematocyst is eGFP negative (arrows). Green, eGFP protein; red, actin filaments; scale bar, 15 μm.

To functionally characterize the putative regulatory sequence of *nb001 in vivo*, we have generated transgenic polyps that express enhanced GFP (eGFP) under the control of the isolated *nb001 *5' flanking sequence. The transgenic construct was made by placing the 1,035 bp *nb001 *promoter (-305 to -1274 relative to the transcription initiation site and including the signal peptide of *nb001*) in front of the GFP reporter gene (Figure 10a). The plasmid was injected into *Hydra *embryos as described [24]. Embryos hatched within 2-3 weeks after injection. Figure 10 shows examples of such transgenic polyps and demonstrates that the 1035 bp 5' flanking region of the *nb001 *gene is able to direct the expression of eGFP in differentiating nematocytes in a pattern that recapitulates precisely the endogenous expression pattern of the *nb001 *gene (see Figure 2 for comparison). Stereo- and confocal microscopy (Figure 10b-f) shows nests of nematocytes with eGFP in groups of 4, 8 and 16 along the body column. This provides *in vivo *proof for the view [25-28] that differentiating nematocytes undergo several rounds of synchronous cell division and remain connected to each other by cytoplasmic bridges prior to terminal differentiation. The *nb001 *gene has a signal peptide, which was included in the construct (Figure 10a). Figure 10c-f shows that the signal peptide drives the eGFP reporter protein into the lumen of the secretory vesicle within differentiating nematocytes. In control transgenic *Hydra *expressing eGFP in nematocytes driven by the *Hydra *actin promoter without a signal peptide, the reporter protein is localized in the cytoplasm (Figure 10g). These results identify the 1035 bp as essential and sufficient for *nb001 *expression *in vivo*.

## Discussion

One of the main challenges in evolutionary biology is to identify the molecular changes that underlie phenotypic differences that are of evolutionary significance [29]. Our results suggest that taxonomically restricted genes are involved in the evolution of morphological novelties such as the cnidarian nematocyst.

### The nematocyte, a cnidarian invention, expresses cnidarian-specific genes

The nematocyte is a cell type exclusively restricted to cnidarians and - from an evolutionary perspective - is considered a neuronal sensory cell [30-32]. During evolution, these neuron-like cells obviously became highly diverged and acquired new cytological features such as the nematocysts (capsules). Each nematocyst consists of an inner and outer capsule wall, an inverted tubule armed with long arrays of spines, and an operculum (for a recent review, see [4]). Development of this cnidarian-specific structure requires complex genetic machinery, consisting of at least two sets of proteins, regulatory transcription factors and structural proteins. One of the few transcription factors identified up to now as being involved in nematocyte differentiation, *Hyzic*, is a homolog of the Zn-finger transcription factor gene *zic*/*odd*-*paired*. *Hyzic *is expressed in the early nematocyte differentiation pathway [32] and may act before, and possibly directly upstream of, *Cnash*, a homolog of the proneural basic helix-loop helix transcription factor gene *achaete*-*scute*.

In contrast to these conserved transcription factors, the downstream structural proteins responsible for putting the nematocysts into shape appear to belong to the group of taxonomically restricted genes. Some of them, such as some minicollagens, spinalin and NOWA, have been reported previously [5,8,33]. Interestingly, in addition to nematocysts, novel proteins appear also to be essential components of other structures of the nematocyte, such as the cnidocil, a cnidarian-specific mechanosensory ciliary structure acting as a 'trigger' for discharge of the nematocyst capsule. The central core of the cnidocil contains a protein, nematocilin, that lacks homologues outside *Hydra *[34]. Two paralogous sequences of nematocilin are present in the *Hydra *genome and appear to be the result of recent gene duplication. Nematocilin is absent in the anthozoan *Nematostella vectensis*; it seems, therefore, to be a gene restricted to the class Hydrozoa.

Nematocysts arguably are one of the most complex secretory products produced by an animal cell [35]. How the different nematocyst morphologies evolved is unknown. David and co-workers [4] have proposed that a diverse set of minicollagen proteins together with a disulfide-linked network of not yet identified fiber-like structures could have been instrumental in the evolution of the different nematocyst morphologies. Our discovery of striking complexity of nematocyte-specific genes at both the genomic and transcriptomic levels may indicate that bundles of protein variants produced by alternative splicing (Figures 4 and 5) and transcription at multiple loci (Figures 6 and 7) contribute to the conformational and structural flexibility of the nematocyst.

Alternative splicing has been proposed as the primary driver of the evolution of phenotypic complexity in mammals [36-38]. While alternative splicing is known to affect more than half of all human genes [38], it has been unclear whether and to what extent a similar mechanism operates in early branching metazoans. Our finding of numerous splice variants in *Hydra*, therefore, was surprising and points to a strong conservation of splicing regulation throughout animal evolution.

Taken together, as described here and consistent with previous studies [5,8,33], the majority of genes encoding nematocyst components have no homologues in higher metazoans and are unique to the cnidarian lineage.

### Transgenic *Hydra *contribute to understanding regulatory evolution and transcriptional control of TRGs

The finding that the differentiation of a taxon-specific cell type, the nematocyte, involves the expression of taxon-specific genes promises to unveil novel aspects of the evolution of this complex cell type in particular and of species-specific traits in general. The work also raises an important question: how do these novel genes interact with upstream transcriptional regulators? Do they contain binding sites for conserved transcription factors? Or do they require novel transcription factors? We have previously hypothesized [12] that taxon-specific genes in combination with the rewiring of the genetic networks of conserved regulatory genes accomplish specification of cnidarian morphologies. Here, in order to address this question experimentally, we took advantage of the recent development of transgenic techniques by embryo-microinjection [24], which offers a rich opportunity to expand research activities in *Hydra *[13,39-41]. As expected, transgenic *Hydra *appear to yield usable insight into the regulatory network controlling expression of genes that lack sequence similarity to known genes. According to the functional analysis of the *nb001 *promoter (Figure 10), the transcriptional machinery regulating TRG expression may involve not yet identified transcription factors. Alternatively, regulatory elements for conserved transcription factors may be highly diverged in promoters of TRGs and, therefore, not detectable in the present approach. Current efforts are directed towards identification of transcription factors causally involved in control of TRG expression.

## Conclusions

Taken together, although certainly much remains to be discovered about the role of TRGs in *Hydra*, the observations presented here reaffirm the view [12,13] that taxon-specific genes account for a substantial part of the *Hydra *genome and may be of profound evolutionary significance both in animals that reach back to the beginnings of metazoan life as well as in more complex organisms.

## Materials and methods

### Animals and culture conditions

Experiments were carried out with *H. vulgaris *strain AEP, *H. magnipapillata *strain 105, and *H. magnipapillata *strain sf1. Transgenic animals were generated using *H. vulgaris *strain AEP [24]. Animals were cultured according to standard procedures at 18°C.

### Supression subtractive hybridization and cDNA library construction

For SSH, double-stranded cDNA was synthesized using 2 μg of mRNA from the temperature sensitive mutant *H. magnipapillata *sf1. SSH was performed using PCR-Select™ cDNA Subtraction kit (Clontech, Mountain View, CA, USA) according to the manufacturer's protocol. Two RNA pools were used for subtractive hybridization (Figure 1). Tester double-stranded cDNA was synthesized from mRNA isolated from heat shocked animals free of i-cells and their derivatives. Driver double-stranded cDNA was synthesized from mRNA from untreated polyps containing all cell types. cDNAs were cloned into pGEM-T vector (Promega, Madison, WI, USA) and transformed into DH5 α *Escherichia coli *cells. Bacterial clones were picked into 384 well plates using Q-Pix roboter and plasmid inserts were sequenced at the Washington University Genome Sequencing Centre (St Louis, MO, USA). Raw sequences were submitted to NCBI dbEST database ([GenBank:CO371734-CO372031], [GenBank:CO373914-CO377781], [GenBank:CO508771-CO510748]).

### Gene expression analysis

To analyze gene expression, whole mount *in situ *hybridization was carried out as described previously [42]. Whole mount double *in situ *hybridization was performed using DIG- and Biotin-labeled RNA probes simultaneously. Antibody incubation and substrate reactions were carried out consecutively as described previously [43]. NBT/BCIP- and Fast Red substrates were used for probe detection according to the manufacturer's instructions (Roche, Nutley, NJ, USA). Riboprobes were prepared with the Dig- and Biotin- RNA labeling kit according to the manufacturer's instructions (Roche).

### Northern blotting

RNA-electrophoresis, transfer, probe-labeling, hybridization and detection procedures were carried out according to standard protocols. For primer sequences used for probe amplification, see Additional data file 1.

### Access to primer and sequence data

For primer sequences used to amplify full-length sequences and splice variants, see Additional data file 1. For retrieval of sequence data and EST contigs, see Additional data file 2.

### Generation of transgenic *H. vulgaris *AEP expressing *nb001*:eGFP

Transgenic founder polyps expressing eGFP under control of the *nb001 *promoter were produced at the University of Kiel Transgenic *Hydra *Facility [44]. The transgenic construct was made by placing the 1,035 bp *nb001 *promoter (-1,075 to +65 relative to the transcription initiation site and including the signal peptide of *nb001*) in front of the reporter gene for eGFP (Figure 10a). The resulting plasmid ligAB was injected into *Hydra *embryos as described [24]. Out of 64 injected embryos, 21 (32%) hatched, from which two lines contained eGFP-positive nematocytes and no eGFP expression in any other cell type. Initial founder transgenic animals were expanded into a mass culture by clonal propagation by budding.

### Microscopy analysis

Fluorescent images were taken on a Zeiss Axioscope fluorescence microscope with an Axiocam (Zeiss) digital camera. Confocal laser microscopy was done using a LEICA TCS SP1 CLS microscope. A Zeiss S420 microscope was used for scanning electron microscopy.

## Abbreviations

EGFP: enhanced GFP; EST: expressed sequence tag; GFP: green fluorescent protein; NCBI: National Centre for Biotechnology Information; SSH: suppression subtractive hybridization; TRG: taxonomically restricted gene; UTR: untranslated region.

## Authors' contributions

SM, GH, and KK designed and carried out the experiments; FAE carried out the confocal microscopy analysis; JW carried out embryo microinjection; TB conceived of the study and participated in its design and coordination; SM, GH, KK, and TB drafted, read and approved the final manuscript.

## Additional data files

The following additional data are available with the online version of this paper. Additional data file 1 is a table listing all primer sequences used to amplify full length sequences and splice variants of the described *Hydra *TRGs. Additional data file 2 is a table showing all GenBank accession numbers of full-length sequences and splice variants and sequence IDs for retrieval of EST contig sequences at [45].

## Supplementary Material

Additional data file 1 Primer sequences used to amplify full length sequences and splice variants of the described *Hydra *TRGs. Click here for file

Additional data file 2 GenBank accession numbers of full-length sequences and splice variants and sequence IDs for retrieval of EST contig sequences at [45]. Click here for file
