# Supplementary material for: Characterization of taxonomically restricted genes in a phylum-restricted cell type
Source: Genome Biol. 2009 Jan 22;10(1):R8. doi: 10.1186/gb-2009-10-1-r8 (PMC2687796; doi:10.1186/gb-2009-10-1-r8)
Supplement: Additional data file 1 — Primer sequences used to amplify full length sequences and splice variants of the described Hydra TRGs. [file gb-2009-10-1-r8-S1.doc]

**Supplementary Table 1:** Primer sequences used to amplify full length sequences and splice variants of the described *Hydra* TRGs; ORF= open reading frame.

| nb-gene | amplified region | primer sequence, forward | primer sequence, reverse |
| --- | --- | --- | --- |
| nb001 | ORF | GGATACAAAACTTGTGGTTCTAGTC | TCTAATGCTTCTTCTGTGGACAAC |
| nb001 | northern-probe | ACGGCTTCACTTGAGCATCT | CTTGTGGTCCTGGCATACCT |
| nb012a | ORF | CTTAAGCGTGAAAGCAAACG | TTTGAATAATCGAGCATTTTG |
| nb012a | northern-probe | GGACTCATGTTGCTGGGACT | ATAGCGCTTCGACGTTGTTG |
| n012b | ORF, partial | CAAAATTATAAATGATAAATCAGG | CTTGTATACTAAGGTTCTG |
| nb012b | northern-probe | CAAAATTATAAATGATAAATCAGG | CTTGTATACTAAGGTTCTG |
| nb035 | ORF | TTGGCACTTCATAGAACAGCG | CTACAGTATGTCTCAAAATTGGATTCTC |
| nb035-sv1 | northern-probe | CGGGTCTAGGCTCAATTCAA | GTCGTCCGTATCATCCTCGT |
| nb035-sv2 | northern-probe | CCAGCTCATGTGCCTGCT | GCCACCACCAGCATAACC |
| nb035-sv3 | northern-probe | GCTGGTAGCTGGGGACAAC | CACCATATCCGCCACCAC |
| nb039a | ORF | CAAGTAGCGGACATCATGAAG | GTTGATCCTTTCTTTTCTTTTGTC |
| nb039a | ORF-5’ to exon4 | CAAGTAGCGGACATCATGAAG | GTGGCTGATCCTTCTTCTTCC |
| nb039a | ORF-5’ to exon7 | CAAGTAGCGGACATCATGAAG | CTCTCCTGATTCAGCATCTTC |
| nb039a | exon4 to exon7 | GGAAGAAGAAGGATCAGCCAC | CTCTCCTGATTCAGCATCTTC |
| nb039b | ORF-5’ to exon4 | GATGAGCCGTTGCAACTGG | CCGTACCAGTGGCTGATC |
| nb039b | ORF to exon5-5’ | GATGAGCCGTTGCAACTGG | CCGTACCAGTGGCTGATC |
| nb039b | exon5-5’ to exon5-3’ | GATCAGCCACTGGTACGG | GGCATCTAAAACAACCATTAGTTTC |
| nb054 | exon3 to ORF-3’ | GACAAGCTGCTCATCATGCC | GTTCGGTAATTTTCGAACTGCATTC |
| nb054 | exon4 to ORF-3’ | GAAGATGTTAAAGTAGAGACTCATG | GTTCGGTAATTTTCGAACTGCATTC |
| nb054 | exon5 to ORF-3’ | GTATCCATCATGAACATGGTGGAC | GTTCGGTAATTTTCGAACTGCATTC |
| nb054 | exon6 to ORF-3’ | GTAGTGATGAAGACATGCCAAATG | GTTCGGTAATTTTCGAACTGCATTC |
| nb054 | northern-probe | CCTGCTCCAAACTTTAAACCTTTAG | GTTCGGTAATTTTCGAACTGCATTC |
| spinalin | northern-probe | ATCGCACAGGCTGCTTTAGT | CATGGTGTCCATGTCCGTAA |
