# Supplementary material for: Characterization of taxonomically restricted genes in a phylum-restricted cell type
Source: Genome Biol. 2009 Jan 22;10(1):R8. doi: 10.1186/gb-2009-10-1-r8 (PMC2687796; doi:10.1186/gb-2009-10-1-r8)
Supplement: Additional data file 2 — GenBank accession numbers of full-length sequences and splice variants and sequence IDs for retrieval of EST contig sequences at [45]. [file gb-2009-10-1-r8-S2.doc]

**Supplementary Table 2:** Genbank accession numbers of full-length sequences and splice variants and sequence IDs for retrieval of EST contig sequences at http://www.compagen.org.

| **nb-gene / TRG** | ***Hydra* species** | **splice variant** | **Compagen ID in dbCAP3_HMAG_051019** | **GenBank accession #** |
| --- | --- | --- | --- | --- |
| *nb001* | *H. mag.* | nb001-sv1 | CL1Contig494 | EU170504 |
| *nb001* | *H. mag.* | nb001-sv2 | CL1Contig291 |  |
| *nb001* | *H. mag.* | nb001-sv3 | CL1Contig106 |  |
| *nb001* | *H. mag.* | nb001-sv4 | CL1Contig86 |  |
| *nb001* | *H. mag.* | nb001-sv5 | CL1Contig659 |  |
| *nb001* | *H. mag.* | nb001-sv6 |  | FJ156099 |
| *nb001* | *H. mag.* | nb001-sv7 |  | FJ156100 |
| *nb001* | *H. mag.* | nb001-sv8 |  | FJ156101 |
| *nb001* | *H. mag.* | nb001-sv9 |  | FJ156102 |
| *nb012a* | *H. mag.* |  | CL243Contig1 | FJ236863 |
| *nb012b* | *H. mag.* |  | CL4997Contig1 | FJ236864 |
| *nb031* | *H. mag.* |  |  | EU015879 |
| *nb035* | *H. mag.* | nb035-sv1 | CL1Contig431 | EU015880 |
| *nb035* | *H. mag.* | nb035-sv2 | CL1Contig609 | FJ177032 |
| *nb035* | *H. mag.* | nb035-sv3 | CL1Contig10 | FJ177033 |
| *nb039a* | *H. mag.* | nb039a-sv1 | CL1Contig99 |  |
| *nb039a* | *H. mag.* | nb039a-sv2 | CL1Contig99 |  |
| *nb039a* | *H. mag.* | nb039a-sv3 | CL1Contig367 |  |
| *nb039a* | *H. mag.* | nb039a-sv4 | CL1Contig423 |  |
| *nb039a* | *H. mag.* | nb039a-sv5 |  | FJ200200 |
| *nb039a* | *H. mag.* | nb039a-sv6 |  | FJ200201 |
| *nb039a* | *H. mag.* | nb039a-sv7 |  | FJ200202 |
| *nb039a* | *H. mag.* | nb039a-sv8 |  | FJ200203 |
| *nb039a* | *H. mag.* | nb039a-sv9 |  | FJ200204 |
| *nb039a* | *H. mag.* | nb039a-sv10 |  | FJ200205 |
| *nb039a* | *H. mag.* | nb039a-sv11 |  | FJ200206 |
| *nb039a* | *H. mag.* | nb039a-sv12 |  | FJ200207 |
| *nb039a* | *H. mag.* | nb039a-sv13 |  | FJ200208 |
| *nb039a* | *H. mag.* | nb039a-sv14 |  | FJ200209 |
| *nb039a* | *H. mag.* | nb039a-sv15 |  | FJ200210 |
| *nb039b* | *H. mag.* |  | CL1Contig99 | FJ196704 |
| *nb039b* | *H. mag.* |  | CL9321Contig1 | FJ196705 |
| *nb039b* | *H. mag.* |  | CL1Contig442 | FJ196706 |
| *nb039b* | *H. mag.* | 039b-sv1 |  | FJ196704 |
| *nb039b* | *H. mag.* | 039b-sv2 |  | FJ196705 |
| *nb039b* | *H. mag.* | 039b-sv3 |  | FJ196706 |
| *nb042* | *H. mag.* |  |  | EU170505 |
| *nb054* | *H. mag.* |  | CL1Contig739 |  |
| *nb054* | *H. mag.* | nb054-sv1 |  | FJ205481 |
| *nb054* | *H. mag.* | nb054-sv2 |  | FJ205482 |
| *nb054* | *H. mag.* | nb054-sv3 |  | FJ205483 |
| *nb054* | *H. mag.* | nb054-sv4 |  | FJ205484 |
| *nb054* | *H. mag.* | nb054-sv5 |  | FJ205485 |
| *nb054* | *H. mag.* | nb054-sv6 |  | FJ205486 |
| *nb054* | *H. mag.* | nb054-sv7 |  | FJ205487 |
| *nb054* | *H. mag.* | nb054-sv8 |  | FJ205488 |
| *nb054* | *H. mag.* | nb054-sv9 |  | FJ205489 |
| *spinalin* | *H. mag.* |  |  | AF043907 |
| *nb082* | *H. mag.* |  |  | FJ222238 |
| *nb001* | *H.oli* |  |  | FJ531796 |
| *nb012a* | *H.oli.* |  |  | FJ232913 |
| *nb035* | *H.oli.* |  |  | FJ232914 |
| *nb039* | *H.oli.* |  |  | FJ232915 |
| *nb042* | *H.oli.* |  |  | FJ232916 |
| *nb054* | *H.oli.* |  |  | FJ232917 |
